# Comparison of nursing diagnostic accuracy when aided by Knowledge-Based Clinical Decision Support Systems with Clinical Diagnostic Validity and Bayesian Decision Models for psychiatric care plan formulation among nursing students: a quasi-experimental study

**DOI:** 10.1186/s12912-023-01292-y

**Published:** 2023-04-27

**Authors:** Kuei-Fang Ho, Po-Hsiang Chou, Min-Huey Chung

**Affiliations:** 1 Department of Nursing, Ching Kuo Institute of Management and Health, Keelung City, Taiwan; 2 School of Nursing, College of Nursing, Taipei Medical University, 250, Wuxing Street, Taipei City, 110 Taiwan; 3 Graduate Institute of Networking and Multimedia, National Taiwan University, Taipei, Taiwan; 4 Department of Nursing, Shuang-Ho Hospital, Taipei Medical University, New Taipei City, Taiwan

**Keywords:** Knowledge-based clinical decision support system, Nursing student, Data science applications in education, Human–computer interface

## Abstract

**Background:**

The most suitable and reliable inference engines for Clinical Decision Support Systems in nursing clinical practice have rarely been explored.

**Purpose:**

This study examined the effect of Clinical Diagnostic Validity-based and Bayesian Decision-based Knowledge-Based Clinical Decision Support Systems on the diagnostic accuracy of nursing students during psychiatric or mental health nursing practicums.

**Methods:**

A single-blinded, non-equivalent control group pretest–posttest design was adopted. The participants were 607 nursing students. In the quasi-experimental design, two intervention groups used either a Knowledge-Based Clinical Decision Support System with the Clinical Diagnostic Validity or a Knowledge-Based Clinical Decision Support System with the Bayesian Decision inference engine to complete their practicum tasks. Additionally, a control group used the psychiatric care planning system without guidance indicators to support their decision-making. SPSS, version 20.0 (IBM, Armonk, NY, USA) was used for data analysis. chi-square (χ2) test and one-way analysis of variance (ANOVA) used for categorical and continuous variables, respectively. Analysis of covariance was done to examine the PPV and sensitivity in the three groups.

**Results:**

Results for the positive predictive value and sensitivity variables indicated that decision-making competency was highest in the Clinical Diagnostic Validity group, followed by the Bayesian and control groups. The Clinical Diagnostic Validity and Bayesian Decision groups significantly outperformed the control group in terms of scores on a 3Q model questionnaire and the modified Technology Acceptance Model 3. In terms of perceived usefulness and behavioral intention, the Clinical Diagnostic Validity group had significantly higher 3Q model and modified Technology Acceptance Model 3 scores than the Bayesian Decision group, which had significantly higher scores than the control group.

**Conclusion:**

Knowledge-Based Clinical Decision Support Systems can be adopted to provide patient-oriented information and assist nursing student in the rapid management of patient information and formulation of patient-centered care plans.

## Background

The American Nurses Association [1] describes the nursing process as the essential actions of nursing practice related to delivering holistic and patient-focused care. The NANDA International (NANDA-I) nursing diagnostic system provides a structure for standardizing nursing terminologies, which enables the exchange of information regarding professional judgments, knowledge, perspectives, and experiences among multiple countries or health-care specialties [2–7]. The use of NANDA-I terminology can improve data collection during the performance of nursing tasks, which can enable patient conditions to be quickly and accurately identified, the quality of care to be improved, and compliance with nursing standards to be enhanced [7–9]. In psychiatric nursing care, advances have been made regarding the use of NANDA-I to describe patient characteristics and nursing diagnoses [10–12].

Information technology has enhanced interoperability in and the exchange of medical information for clinical decision-making [13, 14]. A clinical decision support system (CDSS) is a program module that integrates clinical information and generates intelligent recommendations to thereby enhance medical decision-making and improve health-care delivery [15–18]. CDSSs translate evidence-based practices to increase knowledge and formulate nursing care standards that facilitate clinical decision-making before clinical practitioners make diagnostic decisions or apply treatment actions in medical environments [17, 19–22].

Most CDSSs have been designed as tools that assist nurses in completing various tasks in clinical practice [9, 19, 23, 24]. A CDSS can comprise an integrated module within electronic health records that are structured in accordance with the nursing process that provides accurate, evidence-based, and patient-specific recommendations for nursing students and nurses. This enables them to deliver holistic and patient-focused care in their respective clinical specialties [25–29]. A nursing process-CDSS (NP-CDSS) integrates standardized nursing languages (SNLs; e.g. NANDA-I, Nursing Outcomes Classification, and Nursing Intervention Classification) and knowledge-based indicators to provide improved support for nursing decision-making, enable patient care plans to be reasonably developed, and allow for the accurate assessment of nursing processes, thereby leading to effective nursing interventions being implemented and patient-care goals being reached [25, 26, 28, 29].

Knowledge-Based CDSSs (KBCDSSs) comprise 3 principal parts: (1) a knowledge-based database that extracts specific data from a database, which stores data collected from documents; (2) an inference engine used to calculate indicators according to rules of inference; and (3) a communication mechanism that provides practitioners with evidence-based guidelines for problem recognition and knowledge production, thus aiding decision-making [30–32]. By using comprehensive modules that generate clinical practice guidelines and integrate knowledge databases, KBCDSSs have been used to support professionals in improving patient outcomes, care quality, and clinical health-care value [33].

The knowledge-based database in a KBCDSS is generally generated as a set of rules. AT-H Hao, L-P Wu, A Kumar, W-S Jian, L-F Huang, C–C Kao and C-Y Hsu [4] adopted the Delphi method to help nurses make decisions regarding diagnoses. On the basis of conditional probabilities, Bayesian inference from high-quality data has been used to collect information in clinical environments and develop computer-based decision support regarding the probabilistic relationships between diseases and symptoms [34–37]. Psychiatric nursing is a distinct nursing specialization that is key to the provision of evidence-based and advanced mental health nursing care in various environments [12]. In psychiatric nursing, comprehensive, structured descriptions of individual patient characteristics and psychiatry-specific knowledge must be integrated to ensure and enhance the provision of adequate psychiatric nursing care [10–12]. Accordingly, KF Ho, PH Chou, JC Chao, CY Hsu and MH Chung [9] developed a psychiatric KBCDSS (Psy-KBCDSS) that captures knowledge through rules that are based on the Clinical Diagnostic Validity (CDV) model [38] to validate nursing diagnoses. Thus, the content of nursing diagnoses are verified using the CDV model [39–41], and NANDA-I nursing diagnoses are regarded as representative and adequate [7, 42]. The psychiatric KBCDSS is an empirical decision-making support system that aids decision-making and enhances nurses’ abilities to formulate appropriate patient-oriented care plans [9]. The inference engine (e.g. Bayesian decision model, Delphi method, or CDV model) of a CDSS can be used to improve performance in job-related tasks.

The nursing process involves evidence-based concepts; it combines well-defined assessment, diagnosis, planning, and implementation steps with continual evaluations of nursing effectiveness [1]. Assessment is the first step, and it involves the critical procedure of formulating patient-centered care plans. This step requires critical thinking skills and the collection of subjective and objective patient data [43, 44]. N Aydin and N Akansel [43] indicated that nursing students’ lack of confidence in nursing activities may affect their assessments. In clinical practice, nursing students may exhibit frustration, helplessness, and lack of control because they lack support in the learning environment [45]. To help them undertake clinical tasks, make effective and timely judgments, and improve patient safety, nurses and nursing students must develop psychomotor and critical thinking skills and use their knowledge and abilities to correctly apply various nursing techniques [46, 47]. Health information systems can provide technical assistance to help nurses manage information, make clinical judgments, communicate with health-care teams, guide optimal patient-centered care, organize and record nursing processes, and improve workflows [48–51]. Therefore, health information systems should be used to assist nursing students in developing the ability to analyze subjective and objective patient data and formulate patient-centered care plans [52].

In health-care environments, typical care planning systems simply involve the conversion of paper-based records and free-text input in recording nursing diagnoses. Compared with an advanced care planning system, such systems lack validation mechanisms that can solve the patients’ specific health problems. Suitable inference engines for use in clinical nursing practice have rarely been explored. According to reviews of the literature [53–55] on medical education, few studies have explored learning methodologies for strengthening the practical skills of students in psychiatric or mental health nursing. To the best of our knowledge, even fewer studies have established a CDSS that meets the requirements of timeliness, high quality information, and an appropriate information format to increase nursing students’ decision-making knowledge and skills in patient-centered problem solving. In addition, most studies have evaluated user perceptions of CDSSs by using self-developed questionnaires [4, 9] or satisfaction and usability questionnaires [56]. Few studies have thoroughly evaluated the professional acceptance of or satisfaction with health information technology through assessment instruments or theoretical models, such as the Technology Acceptance Model 3 (TAM3) or the integrating service quality with system and information quality (3Q) model [24, 57]. Extensive research has explored user perceptions of using medical information technology [9, 58–62], which has still not been implemented.

The objectives of this study were to (1) determine the effects of the CDV model and Bayesian decision model on the decision-making of nursing students when they are formulating appropriate patient-oriented care plans and to (2) explore the effects of user perceptions and user opinion acceptance on the adoption of research systems. In the present empirical study, a psychiatric KBCDSS was employed using the CDV inference engine developed by KF Ho, PH Chou, JC Chao, CY Hsu and MH Chung [9] to help future professionals formulate patient-centered care plans during their psychiatric or mental health nursing practicum in clinical settings. The diagnostic accuracy of the CDV model and Bayesian decision model in the KBCDSS were compared with that of a typical psychiatric care planning system. We hypothesized that acceptance, satisfaction, and performance in decision-making competency among nursing students using the KBCDSSs would be significantly higher than those of students using the typical psychiatric care planning system. Because the CDV model typically adopted for validating nursing diagnoses was the inference engine in the KBCDSS, we also hypothesized that nursing students using the KBCDSS with the CDV model would have higher levels of acceptance, satisfaction, and performance in decision-making competency than those using the KBCDSS with the Bayesian decision model. Finally, students using the KBCDSS with the Bayesian decision model were hypothesized to have significantly higher levels of acceptance, satisfaction, and decision-making performance than those in the control group.

## Methodology

### Study design

**Fig. 1:**
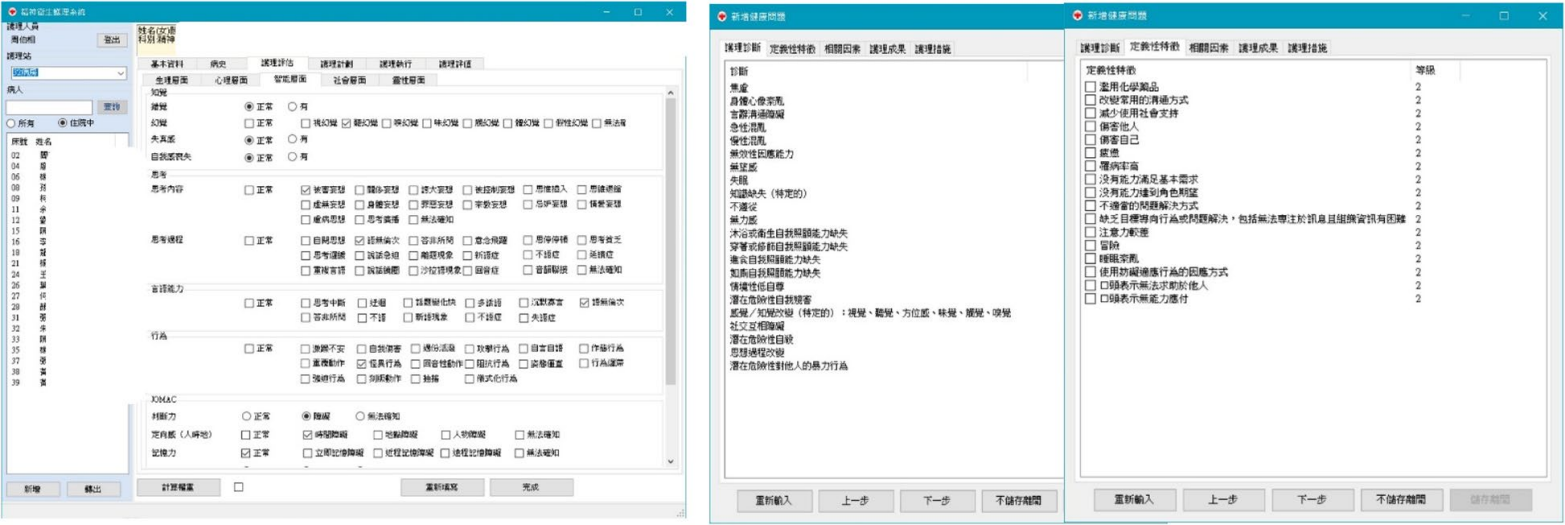
Interface design of the typical psychiatric care planning system used by the control group

**Fig. 2:**
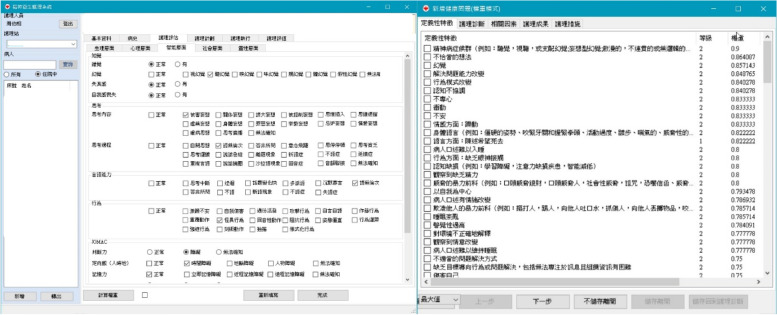
Interface design of the knowledge-based clinical decision support system

A single-blinded, nonequivalent control group design with three groups was employed. Nursing students were allocated to one of the three groups, namely (1) the group using the KBCDSS with the CDV model inference engine (i.e. CDV group), (2) the group using the KBCDSS with the Bayesian Decision model (i.e. BADE group), or the group using the typical psychiatric care planning system (i.e. control group). A quasi-experimental design was used, and the study involved nursing students completing clinical practicums at any time between December 2016 and November 2018. The typical psychiatric care planning system (Fig. 1) was designed based on principles that are commonly recognized to underpin care planning. The CDV-based and Bayesian model-based KBCDSSs (Fig. 2) had a common communication mechanism presented in the user interface, which was based on best practices in the field and featured a combination of the generated evidence-based guidelines database with the unique inference engine. All the participants were blinded to the group allocation. The researchers set the research systems and guidelines for each participant by assigning the participant to an experimental or control group and conducted an individual investigation of specific systems afterward.

### Participants and sample size calculation

All participants were recruited from a single private technology institute located in Northern Taiwan. The nursing students were informed of the proposed activities of the present study during a nursing clinical practicum lecture and invited to participate. The eligible participants were aged 20 years or older, were completing their psychiatric or mental health nursing practicums, had experience with the proposed system, and had voluntarily provided informed consent to participate in the study. After screening, nursing students were excluded from the study if they (1) had never used the proposed system, (2) were not completing the psychiatric or mental health nursing practicums; and (3) had insufficient Chinese language fluency to fully understand and use the assessment instruments.

LV Hedges and EC Hedberg [63] suggested that effect sizes of 0.20 to 0.25 should be considered noteworthy in the field of educational research. Considering baseline imbalance may occur, we select the effect size of 0.20 to be close to 0.14 as the small effect size for the analysis of covariance [64] in a non-randomization design [65]. The sample size was calculated using the G*Power 3.0 software program (UCLA) with three groups, an effect size of 0.20, α of 0.05, power of 0.99, numerator degrees of freedom [*df*] of 2, and 1 covariate. According to a power analysis, the minimum sample size required for each study group was 180. Assuming a dropout rate of 15%, we calculated the requisite minimum total sample size to be 636 (with ≥ 212 participants in each group). In total, 662 nursing students were initially approached to participate, and the responses of 607 students were analyzed, with 55 questionnaires rejected due to incomplete responses; 206, 203, and 198 nursing students were in the control, CDV, and BADE groups, respectively.

### Intervention

Eligible participants were consecutively recruited and allocated to the control, CDV, or BADE groups. For patient condition information, all participants performed comprehensive nursing assessments in accordance with the standards of the Psychiatric Mental Health Nurses' Association of Taiwan [66] and recorded the assessment data into the research systems. The participants in the control group conducted nursing assessments and made diagnoses on the basis of the signs and symptoms displayed by patients; they did so by using a typical psychiatric care planning system and did not receive any guidance. The participants in the CDV group first assessed the signs and symptoms displayed by patients, and they subsequently made individual nursing diagnoses by referencing and screening a list of indicators of patient-specific defining characteristics and related factors or risk factors that were generated and suggested by the CDV inference engine. The participants assigned to the BADE group made nursing diagnoses by applying the practical indicators in the knowledge-based database and using the Bayesian inference engine. After receiving explanations on how to operate the relevant systems, all the participants operated the systems independently to develop individual patient-centered care plans, and they input their results regarding nursing assessment and diagnoses into the system database.

### Information systems design

#### Knowledge-based database framework

To facilitate communication among nurses with various professional specialties in health care, standardized or common languages in nursing environments should be established and promoted to enable effective communication when detailing descriptions of nursing diagnoses, measures, and results [2–4]. SNLs, such as NANDA-I nursing diagnoses, are essential for the successful integration of nursing care records [7, 67] with care planning systems.

The psychiatric care planning system developed by KF Ho, PH Chou, JC Chao, CY Hsu, and MH Chung [9] individually describes each patient within the data repositories of a database; this database incorporates the 5 aspects of the nursing assessment framework established by the Psychiatric Mental Health Nursing Association of Taiwan [66] and NANDA-I nursing diagnoses [7]. This psychiatric care planning system uses a database with 22 nursing diagnoses (based on the NANDA-I framework) that are commonly made in psychiatric wards [9, 11]. The database framework was constructed using nursing assessment variables and diagnoses according to defining characteristics, risk factors, and related factors involved with storing patient-centered care plans. Indicators in the knowledge-based database were generated using the mathematical operations of the CDV inference engine or Bayesian decision model in the operational psychiatric care planning system for each variable.

#### Knowledge database inference engines

##### CDV inference engine

On the basis of a CDV model, KF Ho, PH Chou, JC Chao, CY Hsu and MH Chung [9] developed a KBCDSS comprising a nursing assessment and diagnosis database in the psychiatric care planning system and a CDV inference engine for computing the clinical data of nurses engaging in practical tasks. This KBCDSS is used to support planning and informed decision-making. The system- and evidence-based guidelines provided integrated data that were determined using the frequency of the corresponding nursing assessment items and the defining characteristics or risk factors identified in a knowledge-based module of the Psy-KBCDSS [9]. These relationships are represented by weighted ratios (generated by the nurses’ decision-making processes) for using the psychiatric care planning system in clinical practice.

The CDV model [38] is used to validate nursing diagnoses by using clinical assessments or obtaining clinical information directly from patients, and two expert professionals document observations and ratings [38], identifying and rating items relevant to nursing diagnoses after reaching a consensus regarding the terms considered associated with specific diagnoses. The calculated weighted interrater reliability ratio may provide evidence that practicing nurses can use for diagnosis.

In the CDV model [38], the inference engine calculates the weighted ratios from the association of nursing assessment variables with defining characteristics or related or risk factors for nursing diagnoses. The construction of the formula for the CDV model in the observational approach proceed according to the following steps. (1) Two clinical nurses (a junior clinical nurse and a senior nurse) assess the same patient, and the assessment results, defining characteristics, and related or risk factors associated with the diagnosis are recorded in the database. (2) The frequencies of agreement and disagreement between the 2 nurses’ observations are used to calculate the weighted interrater reliability ratio by using the following KBCDSS_CDV-based formula:

$$R = [(F1/N + F2/N)/2] \times [A/(A + D)],$$

where *F1* is the frequency of nursing assessment variables correlating with defining characteristics and related or risk factors for a nursing diagnosis observed by junior clinical nurses; *F2* is the frequency of nursing assessment variables correlating with defining characteristics and related or risk factors for a nursing diagnosis observed by senior nurses; *N* is the number of patients observed; *A* is the number of instances of agreement; *D* is the number of instances of disagreement; and *R* is the weighted interrater reliability ratio (weighted ratio).

A higher *R* value indicates a higher frequency of agreement between nurses in assessments with defining characteristics and related factors and risk factors for a diagnosis. Weighted ratios of ≥ 0.80 are selected according to the definition of the CDV model [38] to form a list of major weighted ratios, and the level of importance was indicated on the system screen in this study. Weighted ratios of between 0.50 and 0.80 were labeled as minor. Therefore, all the weighted ratios generated by the system were displayed together and guided the nursing students’ decision-making.

##### Bayesian inference engine

In the knowledge-based database of the KBCDSS with the Bayesian decision model, indicators were generated by calculating the associations among the nursing assessment results and the defining characteristics and related or risk factors of diagnoses in clinical practice. We applied the following Bayesian equation:

$$P\left(DC|+\right)=\frac{P\left(+|DC\right)P\left(DC\right)}{P\left(+|DC\right)P\left(DC\right)+P\left(+|Non\_DC\right)P\left(Non\_DC\right)}$$

where *DC* indicates that the occurrence of defining characteristics is true, + indicates that the occurrence of nursing assessment is true, *Non_DC* indicates that the nonoccurrence of defining characteristics is false, *P*( +|*DC*) is the conditional probability that an observed nursing assessment item has 1 specific defining characteristic, *P*(*DC*) is the marginal probability of observing a defining characteristic in all patients, *P*( +|*Non_DC*) is the conditional probability that an observed nursing assessment item does not have 1 specific defining characteristic, *P*(*Non_DC*) is the marginal probability of not observing a defining characteristic for all patients, and *P*(*DC*| +) is the likelihood of 1 defining characteristic being present given that the nursing assessment is accurate. The indicators are obtained by calculating the specific defining characteristic corresponding to each individual nursing assessment item. According to the Bayesian formulation, we also established indicator scores for the relationships of defining characteristics with related factors and the relationships of nursing assessments with risk factors in the knowledge database of the KBCDSS used with the Bayesian decision model.

### Measures

#### Questionnaire for the 3Q model

Technology acceptance and user satisfaction constitute 2 key areas of research on user perceptions of the success of an information system’s implementation [68]. J Xu, I Benbasat and RT Cenfetelli [69] extended the theoretical integration of the concepts of user satisfaction and technology acceptance by BH Wixom and PA Todd [68] to propose a theoretical framework (called the 3Q model) for integrating service quality with system quality and information quality. The 3Q model incorporates user satisfaction, which comprises object-based beliefs (quality of information, systems, and services), object-based attitudes (satisfaction with information, systems, and services), and technology acceptance, which comprises behavioral beliefs (perceived usefulness, ease of use, and enjoyment), behavioral attitude, and intention. To explore users’ perceptions of information systems employed in nursing settings, KF Ho, CH Ho and MH Chung [61] have empirically examined that the 3Q model questionnaire is a valid and reliable means of assessing user beliefs, attitudes, and intentions.

The 3Q model questionnaire is used to collect data on the following variables: (1) object-based beliefs of information quality (i.e. currency, completeness, format, and accuracy), (2) object-based beliefs of system quality (i.e. reliability, accessibility, timeliness, and flexibility), (3) object-based beliefs of service quality (i.e. empathy, service reliability, tangibles, assurance, and responsiveness of the delivered service), (4) object-based attitudes of user satisfaction, (5) behavioral beliefs (i.e. perceived usefulness, perceived ease of use, and perceived enjoyment), and behavioral attitudes and intentions [70]. This survey instrument has 81 items scored on an 11-point Likert scale with endpoints of − 5 (*completely disagree*) and 5 (*completely agree*) and a midpoint of 0 (*neutral*), yielding a total score ranging from − 405 to 405.

The internal consistency of the variables in the 3Q model questionnaire [69] was 0.71 to 0.97, the questionnaire’s composite reliability (CR) was 0.84 to 0.98, and its discriminant validity was satisfactory. KF Ho, CH Ho and MH Chung [61] examined the validity and reliability of the instrument by employing the 3Q model questionnaire to investigate the intentions of nurses to use a care planning system; they found that the instrument had internal consistency reliability (CR = 0.87-0.97 and Cronbach’s α = 0.71-0.95), indicator reliability (all indicator outer loadings > 0.70), and convergent validity (average variance extracted [AVE] = 0.72-0.91). The discriminant validity (the square root of the AVE) exceeded the correlations between constructs, and the model had good fit (standardized root mean residual [SRMR] = 0.056), explaining 53% of the variance in intentions to use the care planning system.

#### Modified TAM3 questionnaire

As noted by M Chuttur [71], the TAM is the most popular model for examining individual reactions to information technology. Both perceived ease of use and perceived usefulness are crucial belief constructs in the original TAM that determine an individual’s behavioral intention in using information technology [72–74]. V Venkatesh and H Bala [72] reviewed research on the TAM and developed the TAM3, which incorporates the determinants of perceived usefulness and perceived ease of use into the original TAM to improve on the TAM2 and on the model containing only the determinants of perceived ease of use.

TAM3 is highly valid, reliable, and accurate when used to predict user perceptions and acceptance from user opinions on the adoption of various information technologies [62]. By exploring nurses’ acceptance of a care planning system, KF Ho, PC Chang, MD Kurniasari, S Susanty and MH Chung [62] identified the determinants of user acceptance and determined the influence of relationships among the variables in the modified TAM3. They also verified that the modified TAM3 is valid and reliable indicator of user acceptance of health information technology in nursing clinical practice.

In the modified TAM3 questionnaire, beliefs are measured using 42 items encompassing the following core concepts: (1) determinants of perceived ease of use, (2) determinants of perceived usefulness, (3) perceived ease of use, (5) perceived usefulness, [70] behavioral intention, (7) moderator (i.e. output quality and voluntariness). In the modified TAM3, the determinants of perceived usefulness comprise subjective norms, image, job relevance, and result demonstrability, and they can explain the association between perceived usefulness and behavioral intention (affected by various determinants). The determinants of perceived ease of use, namely perceptions of external control, computer self-efficacy, computer anxiety, and computer playfulness, and perceived enjoyment, are used to demonstrate the associations between perceived ease of use and its determinants. Accordingly, behavioral intention is determined by perceived usefulness and perceived ease of use. The 4 items of computer self-efficacy are measured on a 10-point Guttman scale ranging from 1 (*strongly disagree*) to 10 (*strongly agree*). Perceptions of the proposed system are assessed using 38 items, with the computer self-efficacy construct excluded, that are scored on a 7-point Likert scale ranging from 1 (*strongly disagree*) to 7 (*strongly agree*).

The original TAM3 questionnaire has high reliability, as indicated by internal consistency (Cronbach’s α = 0.76-0.93), and high validity [72]. The modified TAM3 questionnaire [62] has adequate reliability, as indicated by internal consistency reliability (CR = 0.84-0.96; Cronbach’s α = 0.74-0.94) and indicator reliability (all indicator outer loadings > 0.70), and high validity, as indicated by convergent validity (AVE = 0.64-0.91) and discriminant validity. It also has good model fit (SRMR = 0.09) and accounts for 69% of the total explained variance in intention to use a given information technology system.

#### Performance in decision-making competency among nursing students

In the present study, the researchers were psychiatric nursing teachers with at least a master’s degree and more than 15 years of clinical practice experience; their competence in providing holistic patient care and formulating nursing diagnoses was certified by the Taiwan Nurse’s Association and the Psychiatric Mental Health Nursing Association of Taiwan. To assess the differences in the decision-making competency levels of the nursing students and researchers when they were using the three proposed systems, their decision-making competency was evaluated with respect to its positive predictive value (PPV), sensitivity, and accuracy (true positives, false positives, and false negatives). Thus, the paper-based care plans (baseline data) were compared with the electronic records entered into the databases of the proposed systems (posttest data) by the participants. In both the pretest and posttest phases, the nursing students formulated patient-centered care plans that were validated by the researchers. The subsequent results are expressed in terms of accuracy, PPV, and sensitivity.

To indicate an accurate nursing diagnosis, the students’ decision-making results were required to comply with at least 3 defining characteristics or risk factors [75, 76]. We analyzed the frequency of a nursing diagnosis that was made on the basis of a patient exhibiting at least 3 defining characteristics. Moreover, we analyzed the databases of all the systems and the paper-based care plans to determine the participants’ decision-making competency using the following metrics: (1) the number of cases in which both the nursing student and researcher identified the same defining characteristics (true positives), (2) the number of cases in which the nursing student identified more defining characteristics than the researcher (false positives), (3) the number of cases in which the nursing student identified fewer defining characteristics than the researcher (false negatives), (4) PPV (i.e. true positives/[true positives + false positives]), and (5) sensitivity (i.e. true positives/[true positives + false negatives]).

### Data collection

In accordance with the inclusion criteria, a convenience sample of nursing students were recruited from an institute of technology in Taiwan. Before the intervention, the nursing students were informed of the study in a lecture on the nursing clinical practicum. After the nursing students provided written consent to participate in this study, they were asked to learn and practice the procedures for using the proposed system prior to the actual entry of diagnoses. To minimize missing data, the researchers helped the nursing students use the system and reviewed all the steps involved in establishing patient care plans. During the psychiatric or mental health nursing practicum, we collected the patient care plans formulated by the participants in clinical practice. We obtained paper-based records of case studies and patient care plans from all the nursing students for use as the pretest data.

We numbered the patients and participants, and the nursing students conducted nursing assessments, identified patients’ defining characteristics, and made nursing diagnoses in numerical order across all the three groups. The posttest results in the database represented the defining characteristics and the risk factors identified during the nursing diagnoses produced by the participants when caring for patients in the psychiatric department. The researchers validated the defining characteristic and the risk factor data in the nursing diagnoses from the pretest and posttest phases. Decision-making competency, as defined by true positives, false positives, false negatives, PPV, and sensitivity, was calculated by comparing the pretest and posttest data of the researchers and nursing students in terms of accuracy on defining characteristics. After posttest data collection, all the participants independently completed the questionnaires, which were used to investigate the nursing students’ perceptions of using the proposed system.

### Statistical analysis

All data were analyzed using SPSS, version 20.0 (IBM, Armonk, NY, USA). The homogeneity of sociodemographic variables and the 3 study groups’ usage characteristics for the care planning systems were assessed using descriptive statistics, with the chi-square (*χ*^2^) test and one-way analysis of variance (ANOVA) used for categorical and continuous variables, respectively. A one-way ANOVA was performed to compare the mean 3Q model and modified TAM3 questionnaire scores among the three groups. For this purpose, a post hoc Scheffe’s test was performed if the *F* statistic was significant (*P* < 0.05). A chi-square test was used to analyze the nursing students’ decision-making results for the 3 study groups in the pretest and posttest. A McNemar–Bowker chi-square test was used to determine differences in decision-making competency among the three groups before and after the intervention. We performed analysis of covariance (ANCOVA; with pretest scores controlled as confounders) to examine the PPV and sensitivity in the three groups. A *P* value of < 0.05 was regarded as statistically significant.

## Results

### Baseline characteristics of study participants

**Fig. 3:**
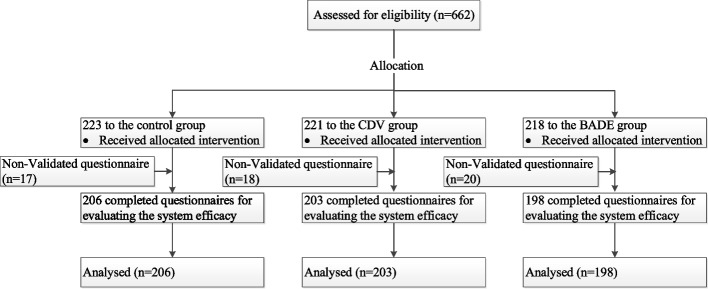
Consolidated Standards of Reporting Trials flow diagram

The general sociodemographic characteristics and baseline outcomes of all the participants are presented in Table 1. The flow the trial is presented in Fig. 3. In total, the data of 607 participants were analyzed. They were divided into the control group (*n* = 206, age 21.30 ± 1.44 years, 95.6% women, 48.5% in 5-year junior college program), CDV group (*n* = 203, age 21.59 ± 1.49 years, 90.1% women, 43.3% in 5-year junior college program), and BADE group (*n* = 198, age 21.53 ± 2.00 years, 90.9% women, 47.5% in 5-year junior college program). Of the participants, 76.6% (465 of 607; 169, 151, and 145 in the control, CDV, and BADE groups, respectively) felt no stress when using a computer. The three groups did not significantly differ in sociodemographic characteristics or baseline outcomes (Table 1). Therefore, the three groups were considered to initially be homogeneous.

**Table 1:** Demographic characteristics and pretest data of the three groups

Demographics and pretest data	Total (*N* = 607)		Control^a^ (*N* = 206)		CDV^b^ (*N* = 203)		BADE^c^ (*N* = 198)		*P* value (χ^2^ or *F*)
	*n*	%	*N*	%	*n*	%	*n*	%	
Age (years), mean (SD)	21.47 (1.66)		21.30 (1.44)		21.59 (1.49)		21.53 (2.00)		.18 (1.74)^j^
Sex									.08 (5.05)
Male	47	7.74	9	4.37	20	9.85	18	9.09	
Female	560	92.26	197	95.63	183	90.15	180	90.91	
Education program									.18 (6.23)
5-year junior college program	282	46.46	100	48.54	88	43.35	94	47.47	
4-year technical program	192	31.63	56	27.18	77	37.93	59	29.80	
2-year technical program	133	21.91	50	24.27	38	18.72	45	22.73	
Weekly computer uses									.08 (11.40)
≤ 3 times	262	43.16	76	36.89	87	42.86	99	50.00	
4 times	188	31.97	79	38.35	61	30.05	48	24.24	
5 times	64	10.54	23	11.17	21	10.34	20	10.10	
≥ 6 times	93	15.32	28	13.56	34	16.75	31	15.66	
Experience stress when using a computer?									.07 (5.21)
Yes	142	23.39	37	17.96	52	25.62	53	26.77	
No	465	76.61	169	82.04	151	74.38	145	73.23	
Compliance with NANDA-I suggestions^d^	438	72.16	144	69.90	152	74.88	142	71.72	.53 (1.29)
False positives^e^	128	21.09	44	21.36	47	23.15	37	18.69	.55 (1.22)
False negatives^f^	305	50.25	104	50.49	95	46.80	106	53.54	.40 (1.83)
True positives^g^	174	28.67	58	28.16	61	30.05	55	27.78	.86 (0.29)
Positive predictive value^h^	0.58		0.57		0.56		0.60		.88 (0.13)^j^
Sensitivity score^i^	0.36		0.36		0.39		0.34		.65 (0.43)^j^

^a^Control: control group using the psychiatric care planning system

^b^CDV, group using the knowledge-based clinical decision support system (KBCDSS) with the clinical diagnostic validity inference engine

^c^BADE, group using the KBCDSS with the Bayesian decision model inference engine

^d^Compliance with NANDA-I suggestions, the frequency with which defining characteristics or risk factors were identified by the participants in accordance with NANDA-I suggestions

^e^False positives, higher frequency of participants identifying defining characteristics than that of the researcher

^f^False negatives, lower frequency of participants identifying defining characteristics than that of the researcher

^g^True positives, equal frequency of participants and the researcher of identifying defining characteristics

^h^Positive predictive value = true positives/(true positives + false positives)

^i^Sensitivity = true positives/(true positives + false negatives)

^j^One-way analysis of variance, *P* value followed by the *F* value in parentheses

### Participants’ perceptions of the intervention

Table 2 presents the 3Q model questionnaire results. One-way ANOVA results indicated significant differences among the three groups in terms of the nursing students’ perceptions after they used the proposed systems. Scheffe’s post hoc test results suggested that the CDV and the BADE groups scored significantly higher than the control group on the criteria of completeness, tangibles, and service quality. On all but the aforementioned variables, the CDV group had the highest mean scores on the scales, followed by BADE and control groups, and the differences among the groups were statistically significant.

**Table 2:** Between-group comparison of posttest scores on the questionnaires inquiring into service, system, and information quality

Construct	Quality dimensions	Control^a^ Mean (SD)	CDV^b^ Mean (SD)	BADE^c^ Mean (SD)	*F* value	*P* value	Scheffe’s test
Information quality		2.01 (1.39)	2.93 (1.35)	2.57 (1.38)	23.38	< .001	a < c < b
	Currency	2.06 (1.35)	2.87 (1.31)	2.51 (1.37)	18.81	< .001	a < c < b
	Completeness	2.22 (1.40)	3.04 (1.32)	2.78 (1.26)	20.65	< .001	a < b; a < c
	Accuracy	1.19 (1.33)	2.57 (1.34)	2.23 (1.24)	18.66	< .001	a < c < b
	Format	2.22 (1.41)	3.09 (1.32)	2.75 (1.28)	22.04	< .001	a < c < b
System quality		2.05 (1.47)	3.07 (1.37)	2.64 (1.42)	26.47	< .001	a < c < b
	Reliability	2.01 (1.33)	2.95 (1.38)	2.53 (1.42)	23.67	< .001	a < c < b
	Accessibility	1.79 (1.51)	2.57 (1.34)	2.23 (1.24)	18.66	< .001	a < c < b
	Flexibility	2.01 (1.36)	3.02 (1.33)	2.63 (1.33)	29.97	< .001	a < c < b
	Timeliness	1.86 (1.31)	3.06 (1.21)	2.66 (1.25)	48.35	< .001	a < c < b
Service quality		2.03 (1.50)	3.02 (1.41)	2.74 (1.34)	26.42	< .001	a < b; a < c
	Responsiveness	2.02 (1.42)	2.99 (1.34)	2.64 (1.48)	24.51	< .001	a < c < b
	Empathy	1.97 (1.41)	2.97 (1.37)	2.63 (1.34)	28.71	< .001	a < c < b
	Service Reliability	2.20 (1.39)	3.11 (1.28)	2.66 (1.38)	23.08	< .001	a < c < b
	Tangibles	1.96 (1.36)	2.87 (1.34)	2.73 (1.34)	27.17	< .001	a < b; a < c
	Assurance	1.93 (1.43)	3.17 (1.29)	2.71 (1.38)	42.14	< .001	a < c < b
User satisfaction		2.04 (1.54)	3.00 (1.39)	2.60 (1.37)	24.15	< .001	a < c < b
Perceived enjoyment		1.80 (1.36)	2.94 (1.36)	2.61 (1.33)	38.55	< .001	a < c < b
Perceived ease of use		1.03 (1.36)	2.80 (1.27)	2.47 (1.27)	107.64	< .001	a < c < b
Perceived usefulness		1.31 (1.35)	3.03 (1.22)	2.60 (1.23)	101.71	< .001	a < c < b
Behavioral attitude		2.24 (1.36)	3.18 (1.29)	2.76 (1.22)	27.06	< .001	a < c < b
Intention		2.36 (1.51)	3.33 (1.35)	2.88 (1.38)	24.05	< .001	a < c < b

^a^Control, control group using the psychiatric care planning system

^b^CDV, group using the knowledge-based clinical decision support system (KBCDSS) with a clinical diagnostic validity inference engine

^c^BADE, group using the KBCDSS with a Bayesian decision model inference engine

The modified TAM3 questionnaire results are presented in Table 3. The one-way ANOVA revealed significant differences among the groups in acceptance levels after using the proposed systems. Scheffe’s post hoc test indicated that scores for computer self-efficacy, computer-associated playfulness, perceived ease of use, perceived usefulness, and behavioral intention were the highest in the CDV group, followed by the BADE and control groups (*P* < 0.05). Additionally, the CDV and BADE groups significantly outperformed the control group on scores for determinants of perceived usefulness (image, job relevance, output quality, and result demonstrability), perceptions of external control, perceived enjoyment, and voluntariness.

**Table 3:** Comparison of the posttest modified technology acceptance model 3 questionnaire scores across the 3 groups

Acceptance scale	Control^a^ Mean (SD)	CDV^b^ Mean (SD)	BADE^c^ Mean (SD)	*F* value	*P* value	Scheffe’s test
Determinants of perceived usefulness						
Subjective norms	4.68 (0.86)	5.11 (0.83)	4.88 (0.82)	13.74	< .001	a < c < b
Image	4.26 (1.02)	4.68 (0.92)	4.57 (0.98)	10.25	< .001	a < b; a < c
Job relevance	4.89 (0.86)	5.34 (0.78)	5.17 (0.79)	15.70	< .001	a < b; a < c
Output quality	4.70 (1.00)	5.22 (0.83)	5.06 (0.85)	18.04	< .001	a < b; a < c
Result demonstrability	4.61 (0.73)	5.07 (0.82)	4.88 (0.82)	17.04	< .001	a < b; a < c
Determinants of perceived ease of use						
Perception of external control	4.79 (0.72)	5.11 (0.69)	4.97 (0.69)	10.49	< .001	a < b; a < c
Computer self-efficacy	6.00 (0.68)	6.35 (0.68)	6.18 (0.68)	13.71	< .001	a < c < b
Computer anxiety	3.05 (0.49)	2.37 (0.57)	2.59 (0.56)	83.77	< .001	b < c < a
Computer playfulness	4.52 (0.67)	4.88 (0.68)	4.69 (0.70)	14.04	< .001	a < c < b
Perceived enjoyment	4.42 (0.81)	4.94 (0.85)	4.72 (0.90)	19.23	< .001	a < c < b
Voluntariness	4.48 (0.87)	4.94 (0.80)	4.76 (0.82)	16.44	< .001	a < b; a < c
Perceived ease of use	4.81 (0.89)	5.25 (0.81)	5.03 (0.90)	13.50	< .001	a < c < b
Perceived usefulness	5.00 (0.86)	5.64 (0.68)	5.31 (0.77)	34.68	< .001	a < c < b
Behavioral intention	4.97 (0.84)	5.46 (0.85)	5.18 (0.80)	18.11	< .001	a < c < b

^a^Control, control group using the psychiatric care planning system

^b^CDV, group using the knowledge-based clinical decision support system (KBCDSS) with the clinical diagnostic validity inference engine

^c^BADE, group using the KBCDSS with the Bayesian decision model inference engine

### Decision-making competency of nursing students

Table 4 presents the differences in true positives, false positives, false negatives, PPV, and sensitivity among the three groups. The chi-square test revealed that the highest values for compliance with the suggestions of NANDA-I (*P* < 0.001) and true positives (*P* < 0.001) were obtained by the CDV group, followed by those obtained by the BADE and control groups. In the CDV and BADE groups, the nursing students who formulated nursing diagnoses with at least three defining characteristics that were consistent with the suggestions of NANDA-I and the true positive results exhibited significant improvements in their decision-making competency from their baseline; by contrast, no significant difference was detected between the baseline and posttest results of the control group. The CDV and BADE groups exhibited improvements with respect to false positives and false negatives. However, the control group exhibited no improvement with respect to false positives, false negatives, and true positives. According to the ANCOVA results, the three groups significantly improved with respect to PPV (*P* < 0.001) and sensitivity (*P* < 0.001), with PPV and sensitivity having the greatest improvement in the CDV group, followed by the BADE and control groups.

**Table 4:** Pretest and posttest results on nursing students’ decision-making competency

Outcomes	Group	Pretest		Posttest		*P* value
		*n* (%)	*P* value (*x*^2^)	*n* (%)	*P* value (χ^2^)	
Compliance with NANDA-I suggestions^d^	Control^a^	144 (69.90)	.53(1.29)	155 (75.24)	< .001 (29.57) b > c > a	.15^j^
	CDV^b^	152 (74.88)		191 (94.09)		< .001^j^
	BADE^c^	142 (71.72)		172 (86.87)		< .001^j^
False positives^e^	Control^a^	44 (21.36)	.55(1.22)	37 (17.96)	0.01 (8.53) a > b	.12^j^
	CDV^b^	47 (23.15)		17 (8.37)		< .001^j^
	BADE^c^	37 (18.69)		24 (12.12)		.03^j^
False negatives^f^	Control^a^	104 (50.49)	.40(1.83)	106 (51.46)	< .001 (20.72) a > b; a > c	.87^j^
	CDV^b^	95 (46.80)		60 (29.56)		< .001^j^
	BADE^c^	106 (53.54)		76 (38.38)		< .001^j^
True positives^g^	Control^a^	58 (28.16)	.86(.29)	63 (30.58)	< .001 (41.24) b > c > a	.51^j^
	CDV^b^	61 (30.05)		126 (62.07)		< .001^j^
	BADE^c^	55 (27.78)		98 (49.49)		< .001^j^
Positive predictive value^h^	Control^a^	0.57		0.63		< .001 (*F* = 9.78)^k^ b > c > a
	CDV^b^	0.56		0.88		
	BADE^c^	0.60		0.80		
Sensitivity^i^	Control^a^	0.36		0.37		< .001 (*F* = 23.26)^k^ b > c > a
	CDV^b^	0.39		0.68		
	BADE^c^	0.34		0.56		

^a^Control, control group using the psychiatric care planning system

^b^CDV, group using the knowledge-based clinical decision support system (KBCDSS) with the clinical diagnostic validity inference engine

^c^BADE, group using the KBCDSS with the Bayesian inference engine

^d^Compliance with NANDA-I suggestions, the frequency with which defining characteristics or risk factors were identified by participants in accordance with the suggestions of NANDA-I nursing diagnoses

^e^False positives, higher frequency of participants identifying defining characteristics than that of the researcher

^f^False negatives, lower frequency of participants identifying defining characteristics than that of the researcher

^g^True positives, equal frequency of participants and the researcher of identifying defining characteristics

^h^Positive predictive value = true positives/(true positives + false positives)

^i^Sensitivity = true positives/(true positives + false negatives)

^j^McNemer’s test for within-group differences in proportional variables

^k^Analysis of covariance—*P* values are followed by *F* values in parentheses

## Discussion

### Key findings

To the best of our knowledge, this is the first study to determine the effect of KBCDSSs on the diagnostic accuracy of nursing students in their decision-making when formulating patient-focused care plans; our findings can assist future professionals in honing their knowledge and professional skills in various domains. Our results indicate that the participants who used the KBCDSS with the CDV or Bayesian decision models had higher posttest scores on perceptions of the target system and decision-making competency relative to the control group.

Functional suitability, result demonstrability, currency, completeness, responsiveness, performance efficiency, compatibility, reliability, accessibility, timeliness, usefulness, and ease of use are key factors that affect individual perceptions regarding the adoption of nursing process systems [61, 62, 77]. In this study, the nursing students’ performance and mean acceptance, satisfaction, and behavioral intention scores on the 3Q model and modified TAM3 questionnaires were the highest in the CDV group, followed by the BADE group and the control group, in which a psychiatric care planning system was used without guidance. The participants noted that the CDV inference engine provided empirical knowledge-based guidelines that were complete, reliable, clinically useful, and prompt in their delivery to fill gaps in their critical thinking, enhance their knowledge of the nursing process, and improve their decision-making competency. The nursing students were most satisfied with and most broadly accepted the CDV model. Therefore, we suggest that the CDV model, which was used to verify the content of nursing diagnoses in the KBCDSS, is an innovative, appropriate, and reliable decision model for use in clinical nursing practice. Our results demonstrate that psychiatric KBCDSSs can provide intelligent technologies that assist decision-makers who must rapidly process information and formulate patient-centered care plans accordingly.

### Evaluation of decision-making competency of nursing students

NP-CDSSs with SNLs can support users by providing evidence-based nursing diagnoses, outcomes, and interventions [25–29, 77]. In the present study, the knowledge-based database of the utilized KBCDSSs applied a CDV model or Bayesian decision model to compute patient information with an inference engine, translate evidence-based practices, and emulate the thought process of real-life professionals to provide best practice guidelines for decision-making. The CDV and BADE groups significantly outperformed the control group in terms of sensitivity, PPV, true positives, false positives, and false negatives in the post-test stage (Table 4). The results therefore suggest that participants in the intervention (CDV and BADE) groups exhibited significantly improved decision-making competency in formulating a patient-centered care plan. This was because the weighted ratios in the CDV inference engine and guideline indicators in the Bayesian decision model inference engine (for defining characteristics and risk factors) effectively helped the participants to make informed decisions. This outcome validates the effect of KBCDSSs with CDV and Bayesian inference engines on the diagnostic accuracy of developing appropriate patient-centered care plans.

In the pretest, approximately 30% of the participants identified less than 3 defining characteristics or risk factors in their nursing diagnoses, with the 2 intervention groups and control group not differing significantly in this regard. However, with the adoption of the CDV inference engine or Bayesian decision model in the KBCDSS, the participants not only significantly outperformed the nursing students using the psychiatric care planning system in the posttest but also exhibited significant improvement in their own the pretest and posttest scores. The nursing students experienced difficulties in determining diagnoses and levels of patient health problems in clinical settings by using NANDA-I diagnoses [43]. We contend that the supportive functions of KBCDSSs provide equal levels of support for the planning, decision-making, and implementation phases of patient-centered care plans. Hence, KBCDSSs can supplement conventional nursing education methods by providing guidelines to support the clinical decision-making and operational needs of nursing students.

The three groups significantly differed in terms of their PPVs, sensitivity, true positive outcomes, and rates of compliance with the suggestions of NANDA-I; the CDV group outperformed the BADE group, and the results of the BADE and control groups differed significantly. The construction of a KBCDSS that uses SNL and CDV models as a decision rule for obtaining evidence-based guidelines has produced highly significant empirical results; these guidelines help nurses to make accurate nursing diagnoses and correctly execute nursing procedures [9]. Therefore, we argue that the CDV inference engine translates evidence-based practices into knowledge that comprehensively assists users from all nursing specialties in managing their information and organizing their assessment data.

Generally, nurses must undergo several months of guided clinical reasoning training programs to enhance their nursing competency in performing critical thinking and reflection and in accurately processing information to formulate individualized care plans [78–81]. Computer-based nursing information systems can help nurses make timely decisions and provide accurate, effective, and individualized care to patients [4, 82, 83]. We suggest that the CDV model can be used with machine intelligence in KBCDSSs to help nursing students apply theoretical and practical skills, thereby improving their clinical competency as they gradually assimilate into the nursing environment.

### KBCDSS satisfaction and acceptance

According to the 3Q model questionnaire on user satisfaction and perceived ease of use (Table 2) and to the modified TAM3 questionnaire on the determinants of perceived usefulness and voluntariness (Table 3), the CDV and BADE groups had more positive perceptions of the KBCDSS than the control group. In this study, both the weighted ratios of the CDV inference engine and the guideline indicators of the Bayesian decision model inference engine provided patient-oriented empirical guidelines that helped nursing students to rapidly assess individual patients and formulate appropriate patient-centered care plans. NO Yakovleva and EV Yakovlev [84] demonstrated that modern education methodologies should focus on facilitating self-learning and providing comfortable environments and flexible training programs in which students can exercise their initiative. Thus, the findings of the present study demonstrate that nursing students can use a KBCDSS to access support mechanisms for clinical task–specific needs and utilize educational resources. Our results indicate that students had favorable perceptions of the KBCDSS as a means to support their decision-making and operational needs in formulating patient-centered care plans. We suggest the use of KBCDSSs as a means of assisting nursing students with clinical decision-making in practicum environments.

In the 3Q model questionnaire, user satisfaction was reported using the themes of object-based beliefs and user satisfaction (Table 2). The CDV group exhibited significantly greater user satisfaction than the BADE group, and user satisfaction in the BADE group differed significantly from that in the control group. Moreover, the acceptance results for the modified TAM3 questionnaire (Table 3) and 3Q model (Table 2) were significantly higher among the CDV group participants. The CDV inference engine calculates weighted ratios by using the CDV model [38]. In the present study, major weighted ratios had values of > 0.80, in accordance with the CDV model of Fehring [38]. Because major weighted ratios were used as assessment criteria for our participants, the nursing students in the CDV group preferred the extraction guidelines of the CDV model’s weighted ratios and exhibited higher acceptance than the students in the other groups did.

Students become more conversant with computers and better keep up with changes in technology the more they use them [85]. Users gain greater computer self-efficacy the more they use computers to manage their daily tasks [86]. Among the participants in this study, 74.2% reported using a computer less than 5 times a week on average and 23.4% reported experiencing stress when using a computer. The nursing students’ perceptions of the determinants of perceived ease of use (computer self-efficacy, computer anxiety, and computer playfulness) on the modified TAM3 questionnaires were the highest in the CDV group, followed by the BADE group and the control group, in which the psychiatric care planning system was used without guidance. This result indicated that participants had the highest acceptance and the lowest stress regarding using the CDV model inference engine with the KBCDSS, even if they had little experience in operating the system.

The participants who used the KBCDSS with the CDV inference engine had significantly higher scores on the 3Q model questionnaire in terms of information quality (currency, accuracy, and format), system quality (reliability, accessibility, flexibility, and timeliness), and service quality (empathy, service reliability, assurance, and responsiveness). To meet the demands of nursing work, future professionals must be able to respond to clinical needs in complex health-care environments [54]. In the present study, the CDV inference engine in the KBCDSS achieved timely provision of the most up-to-date, comprehensive, correct, secure, and highly reliable information to improve performance in various nursing tasks in clinical practice. An appropriate teaching method is one that builds knowledge in a step-by-step manner beginning from what learners already know to cultivate their enthusiasm for learning [87]. The nursing students involved in our study sensed that the CDV model inference engine of the KBCDSS was used to help incrementally hone their skills of formulating appropriate, informative, and easy-to-follow care plans. Therefore, the participants exhibited strong behavioral intentions for using the CDV model inference engine of the KBCDSS. Our findings indicate that the knowledge-based weighted ratios of the CDV model inference engine in the KBCDSS are the most favorable type of support for nursing students striving to develop competency in patient-centered care planning. The results demonstrate that the adoption of reasoning rules, such as those of the CDV model, can be used as a suitable and reliable validation tool in nursing to develop an inference engine for a KBCDSS in clinical nursing practice.

### Limitations and recommendations for future research

This study has several limitations. First, the participants were only recruited from a single institute of technology in Taiwan and were not randomly assigned to groups. Therefore, the findings cannot be generalized to all populations. Future researchers should recruit students from multiple schools in a randomized, blinded, controlled trial. Finally, the outcome variable of behavioral intention was not measured in this study’s pretest phase. Therefore, future studies can measure behavioral intention to evaluate perceptions of the proposed system and compare pretest versus posttest user intentions.

## Conclusions

The CDV and Bayesian inference engines in a KBCDSS supported nursing students’ decision-making (e.g. formulating individual care plans and performing appropriate nursing tasks) during their psychiatric or mental health nursing practicums. This study’s participants were satisfied with and accepted the KBCDSS. The highest satisfaction, acceptance, and performance in decision-making competency were exhibited by the nursing students using the CDV-based KBCDSS. Future KBCDSSs can integrate advanced digital technologies with other decision-making functions and standards in nursing education to develop pedagogical strategies that simulate the realities of health-care environments for improved learning outcomes and greater student engagement.

## Data Availability

The datasets used and/or analyze during the current study are available from the corresponding author on reasonable request.

## Abbreviations

KBCDSS: Knowledge-Based Clinical Decision Support System
CDV: Clinical Diagnostic Validity
BADE: Bayesian Decision
PPV: Positive Predictive Value
